# SARS-CoV-2 protein subunit vaccination of mice and rhesus macaques elicits potent and durable neutralizing antibody responses

**DOI:** 10.1016/j.xcrm.2021.100252

**Published:** 2021-04-05

**Authors:** Marco Mandolesi, Daniel J. Sheward, Leo Hanke, Junjie Ma, Pradeepa Pushparaj, Laura Perez Vidakovics, Changil Kim, Monika Àdori, Klara Lenart, Karin Loré, Xaquin Castro Dopico, Jonathan M. Coquet, Gerald M. McInerney, Gunilla B. Karlsson Hedestam, Ben Murrell

**Affiliations:** 1 Department of Microbiology, Tumor and Cell Biology, Karolinska Institutet, Stockholm, Sweden; 2 Division of Medical Virology, Institute of Infectious Diseases and Molecular Medicine, University of Cape Town, Cape Town, South Africa; 3 Department of Medicine, Solna, Karolinska Institutet and Karolinska University Hospital, Stockholm, Sweden

**Keywords:** SARS-CoV-2, neutralizing antibodies, protein subunit vaccine

## Abstract

The outbreak and spread of SARS-CoV-2 (severe acute respiratory syndrome-coronavirus-2) is a current global health emergency, and effective prophylactic vaccines are needed urgently. The spike glycoprotein of SARS-CoV-2 mediates entry into host cells, and thus is the target of neutralizing antibodies. Here, we show that adjuvanted protein immunization with soluble SARS-CoV-2 spike trimers, stabilized in prefusion conformation, results in potent antibody responses in mice and rhesus macaques, with neutralizing antibody titers exceeding those typically measured in SARS-CoV-2 seropositive humans by more than one order of magnitude. Neutralizing antibody responses were observed after a single dose, with exceptionally high titers achieved after boosting. A follow-up to monitor the waning of the neutralizing antibody responses in rhesus macaques demonstrated durable responses that were maintained at high and stable levels at least 4 months after boosting. These data support the development of adjuvanted SARS-CoV-2 prefusion-stabilized spike protein subunit vaccines.

## Highlights

Neutralizing antibodies are detectable after one adjuvanted spike protein immunization
Boosting yields higher neutralizing titers than observed in seropositive human donors
Neutralizing antibody titers remain high for at least 4 months after the last boost
Immunization stimulates spike-specific memory B cells

## Graphical abstract

**Figure:**
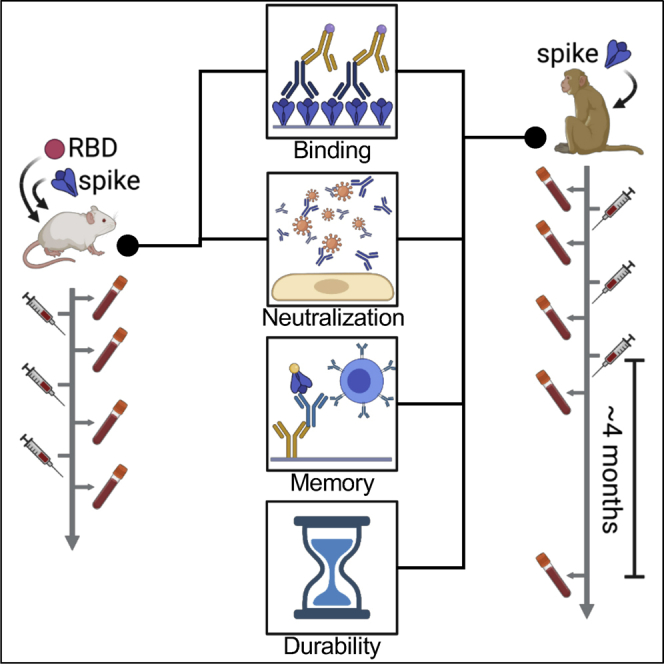

## Teaser

Mandolesi et al. show that immunization with adjuvanted prefusion-stabilized SARS-CoV-2 spike glycoprotein yields potent antibody responses in mice and macaques. Neutralizing antibodies are detectable after one immunization. Boosting results in exceptional potency, with neutralizing titers exceeding (>10-fold) those observed in seropositive humans and remaining high during a 4-month follow-up.

## Introduction

As of February 2021, over 109 million cases of severe acute respiratory syndrome-coronavirus-2 (SARS-CoV-2) infection have been confirmed, with more than 2.4 million coronavirus disease 2019 (COVID-19)-related deaths recorded.^1^ Cases continue to increase globally despite unprecedented public health measures and lockdowns. Effective prophylactic vaccines are urgently required. Adjuvanted recombinant protein subunit vaccines have excellent safety profiles and represent a proven vaccine platform for eliciting protective immune responses to viral infections, including human papillomavirus (HPV), hepatitis B virus (HBV), and influenza A virus.

**Figure 1:**
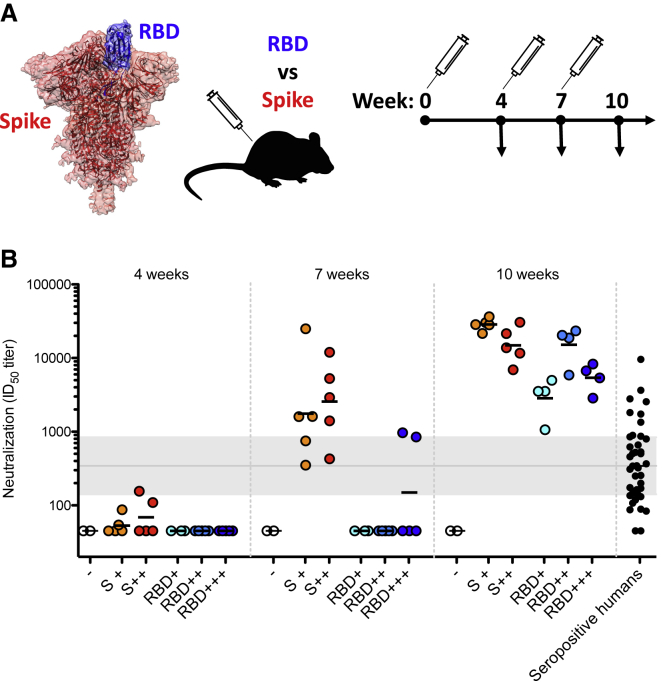
Protein subunit vaccines elicit potent neutralizing antibodies in mice (A) Two SARS-CoV-2 protein immunogens were evaluated: stabilized spike trimer and receptor-binding domain (RBD). Mice (N = 24) were immunized and humoral immune responses were followed longitudinally to compare stabilized spike versus RBD immunogens at several doses. (B) Serial dilutions of serum from immunized mice were assessed for neutralization of SARS-CoV-2 pseudotyped lentiviruses harboring a luciferase reporter gene, and the ID_50_ titers were calculated as the reciprocal dilution where infection (RLU) was reduced by 50% relative to infection in the absence of serum. The geometric mean ID_50_ for each group is displayed. Unimmunized mice, open circles; S+ (5 μg stabilized spike, orange); S++ (25 μg stabilized spike, red); RBD+ (5 μg RBD, cyan); RBD++ (25 μg RBD, blue); RBD+++ (50 μg RBD, navy). ID_50_ titers below the limit of detection (45 or 90 depending on sample availability) are displayed as 45. ID_50_ titers in seropositive donors (black) in Castro Dopico et al.^14^ determined using the same assay, and the median and interquartile range is highlighted in gray across the background.

The spike glycoprotein of SARS-CoV-2 mediates receptor binding and entry into target cells and is the primary target for vaccine design. The receptor-binding domain (RBD) is a stable subunit within the spike glycoprotein (Figure 1A) responsible for angiotensin-converting enzyme 2 (ACE2) binding^2^, ^3^, ^4^, ^5^, ^6^ that can be expressed as an independent domain.^6^, ^7^, ^8^ While the RBD is a major target for neutralizing antibodies,^9^, ^10^, ^11^ antibodies specific for spike epitopes outside the RBD are also capable of neutralization.^12^^,^^13^

To evaluate the use and immunogenicity of recombinant protein subunit vaccines for SARS-CoV-2, we immunized C57BL/6J mice (N = 24) with either a trimeric form of the spike ectodomain or with RBD, produced in 293-F cells. The RBD domain was expressed as an Fc-fusion protein, which was cleaved and the RBD subsequently purified by size-exclusion chromatography. The spike ectodomain was expressed as a prefusion-stabilized variant with a C-terminal T4 trimerization domain and the introduction of two stabilizing proline mutations in the C-terminal S2 fusion machinery.^15^, ^16^, ^17^ Pure, homogeneous spike glycoprotein trimers were obtained by affinity purification followed by size-exclusion chromatography . We previously showed,^18^ using cryoelectron microscopy (cryo-EM), that such spike preparations are well folded and maintained in the trimeric, prefusion conformation, consistent with the original report.^15^

Mice were immunized with varying doses of antigen (range: 5–50 μg) in 50 μL AddaVax (InvivoGen), a squalene-based oil-in-water emulsion analogous to MF59. MF59 is licensed, safe, and effective in humans,^19^ and increases the immunogenicity of an influenza vaccine in the elderly.^20^ Mice were boosted twice, at 3-week intervals, beginning 4 weeks after prime (Figure 1A). In both the low-dose (5 μg) and high-dose (25 μg) groups, a single immunization with prefusion-stabilized spike elicited a strong spike-specific immunoglobulin G (IgG) antibody response, detected by ELISA (Figures S1A and S1B), as early as 4 weeks following the first immunization. RBD was less immunogenic, with weak to no detectable responses after the initial prime. However, seroconversion was evident in all of the mice after the first boost with RBD.

To determine whether the antibody responses elicited were neutralizing, we used a SARS-CoV-2 pseudotyped virus neutralization assay. In spike-immunized mice, neutralizing responses (median infectious dose [ID_50_] ∼100) were already detectable in 4 of 10 mice after a single dose (Figure 1B and S1C–S1F). All spike-immunized mice developed potent neutralizing antibody responses (ID_50_ ∼1,600) after the first boost, which further increased in potency (ID_50_ ∼25,000) after the second boost. In contrast, RBD-immunized mice developed consistent neutralizing antibody responses only after the second boost, with an ID_50_ neutralizing antibody titer across all groups of ∼5,300. The substantial enhancement of the virus neutralizing activity observed after the second RBD boost warrants further investigation. Pooling across doses, neutralizing antibody titers for the spike-immunized mice were significantly higher than those for RBD-immunized mice after both the first (p < 0.01) and second (p < 0.01) boosts. At matched doses, 5 μg spike elicited significantly higher neutralizing antibody titers than 5 μg RBD after the first (p < 0.01) and second (p < 0.01) boosts, and 25 μg spike elicited significantly higher neutralizing antibody titers than 25 μg RBD after the first (p < 0.01) but not after the second boost (p > 0.05). Pooling doses and neutralization titers increased over each immunization for spike (all p < 0.01) and between weeks 7 and 10 for RBD (p < 0.01), but not between weeks 4 and 7 (p > 0.05). Across all of the groups, spike-specific IgG titers correlated strongly with pseudovirus neutralization, although neutralization was detectable only above a threshold half-maximal effective concentration (EC_50_) (Figure S1G).

**Figure 2:**
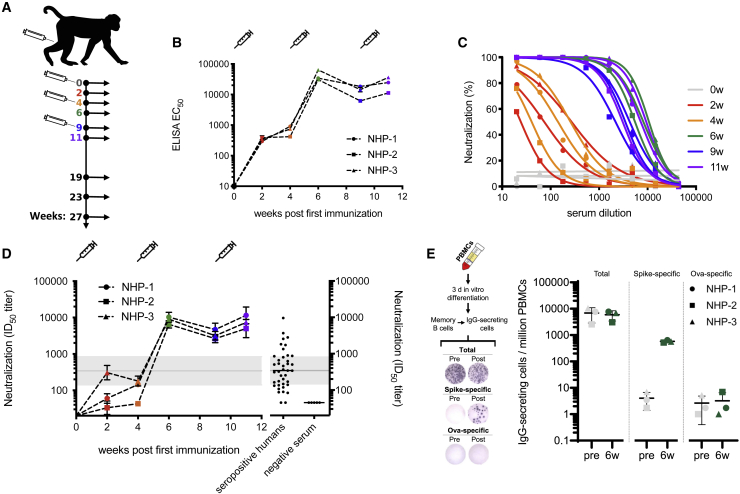
Prefusion-stabilized SARS-CoV-2 spike protein subunit vaccine reproducibly elicits potent neutralizing antibody responses in non-human primates (A) Macaque (N = 3) immunization and sampling schedule. Syringes indicate the timing of immunizations, and arrows denote times at which blood was drawn. (B) Longitudinal spike directed IgG responses. (C) Neutralization curves depicting percent neutralization against serum dilution. (D) Longitudinal neutralization potency up to week 11. Neutralization below the assay limit of detection (20) is plotted as 20, and error bars depict geometric SD about the geometric mean for 3 replicate measurements. Shaded band corresponds to the median and interquartile range of the neutralization potency observed in seropositive human donors using the same assay^14^ shown at right. (E) B cell ELISPOT analysis of *in vitro* differentiated memory B cells 2 weeks after the second immunization, shown as frequency of spike-specific IgG-secreting cells per million PBMCs. Error bars depict standard deviation about the mean.

Next, we immunized three rhesus macaques (*Macaca mulatta*) with adjuvanted trimeric prefusion-stabilized spike glycoprotein over –4-week intervals (Figure 2A) and characterized the titers and kinetics of binding and neutralizing antibodies. Macaques were immunized intramuscularly with 100 μg spike protein in 75 μg Matrix-M (Novavax AB), a saponin-based adjuvant developed for clinical use.^21^ Neutralizing antibody responses were already detectable 2 weeks after a single dose, reaching ID_50_ titers in the range of 90–300 at 4 weeks. Two weeks after a first boost, the neutralizing antibody responses were extremely potent, with ID_50_ titers peaking at ∼10,000 in all 3 macaques. An additional boost 3 weeks later did not raise the peak neutralization potency above that obtained with only 2 immunizations, suggesting diminishing returns of a third spike protein-based dose with this interval after the second injection (Figures 2C and 2D).

A SARS-CoV-2 strain harboring a D614G mutation in the spike protein is prevalent globally.^22^^,^^23^ We examined the neutralizing antibody responses elicited in immunized macaques against the D614G variant and observed neutralizing titers that were comparable those observed against the wild-type (vaccine) strain (Figure S1I).

While a standard neutralizing antibody assay has not been universally adopted, comparisons to a common reference point, such as seropositive human cohorts assayed in the same way, can calibrate titers. After 2 prefusion-stabilized spike protein immunizations, geometric mean ID_50_ neutralizing antibody titers in macaques were >1 order of magnitude higher than those measured in sera from SARS-CoV-2 seropositive humans analyzed within 1 month after a positive PCR test (Figure 2D). The ID_50_ neutralizing antibody titers were also substantially higher than those elicited by other immunization platforms that afforded macaques partial or complete protection from challenge in other studies.^24^^,^^25^

The spike-directed IgG binding titers elicited in the macaques (Figure 2B) correlated strongly with the virus neutralizing activity (R^2^ = 0.9478; Figure S1H). Recent data show that many neutralizing monoclonal antibodies isolated from SARS-CoV-2 convalescent individuals display low levels of somatic hypermutation (SHM),^11^^,^^26^^,^^27^ providing one possible explanation for the rapid development of neutralization in immunized animals. The clonality, antibody germline VDJ usage, and level of SHM that characterizes vaccine-induced SARS-CoV-2 spike-induced antibody responses will be a matter of interest, both in the macaque model and in human vaccine trials.

Reported neutralizing antibody titers have varied substantially across different vaccine platforms. In animal models, inactivated virus, DNA-based vaccines, and adenovirus-vectored vaccines elicited peak neutralizing antibody titers similar to or lower than those seen in convalescent sera.^24^^,^^25^^,^^28^ While high-dose mRNA-based immunizations elicited potent neutralizing antibody responses in mice,^29^ neutralizing antibody titers elicited in Phase I/II human trials were markedly lower.^30^^,^^31^ Immunization of mice with mRNA-encoding membrane-anchored stabilized spike led to reduced or undetectable viral loads in the lungs and nasal turbinates in a dose-dependent manner, and susceptible transgenic mice were protected from lethal challenge.^29^ Other preclinical vaccine studies using nucleic acid platforms elicited immune responses that protected against disease but not against infection. For example, RBD- and spike-encoding DNA vaccines led to reduced viral loads in the nose and lungs of challenged macaques.^24^^,^^25^ Immunizations with recombinant spike protein subunits, including RBD and S1 subunits, elicited strong neutralizing antibody responses^32^, ^33^, ^34^ and could protect non-human primates from infection.^32^

In our study, two inoculations of Matrix-M-adjuvated SARS-CoV-2 spike trimers also generated robust antigen-specific memory B cell responses in all three macaques. Their presence was determined by *in vitro* stimulation of memory B cells into antibody-secreting plasma cells and enumeration by B cell ELISpot analysis, as previously described.^35^ The frequency of total IgG-secreting cells per plated peripheral blood mononuclear cell (PBMC) was similar for pre- and post-vaccination samples. In contrast, SARS-CoV-2 spike-specific IgG-secreting cells were absent in the pre-vaccination samples while spike-specific IgG-secreting cells were clearly present in the post-vaccination samples. Control samples probed with an irrelevant antigen (Ova) were negative at both time points (Figure 2E). These results demonstrate that two inoculations of adjuvanted spike protein established a population of memory B cells capable of differentiation into SARS-CoV-2 spike-specific IgG-secreting plasma cells following re-stimulation.

At least 66 candidate SARS-CoV-2 vaccines are in clinical development, including 18 that have already entered Phase III clinical trials.^36^ These include DNA and RNA-based platforms (Moderna,^30^^,^^37^ Inovio, and BioNTech/Pfizer^38^^,^^39^), adenovirus-vectored vaccines (CanSino,^40^ University of Oxford/AstraZeneca,^41^^,^^42^ Janssen Pharmaceuticals,^43^^,^^44^ and Sputnik V^45^), inactivated virus vaccines (Sinovac,^46^ Wuhan Institute of Biological Products, and Beijing Institute of Biological Products), and recombinant protein vaccines (Novavax,^47^^,^^48^ Anhui Zhifei Longcom Biopharmaceutical, and Clover Biopharmaceuticals/GlaxoSmithKline). Given the urgency in the current pandemic, vaccines require rapid mobilization on a global scale.

Efficacy trials for two mRNA-based vaccines have reported extremely promising results. Two doses of mRNA-1273 (Moderna) administered 28 days apart was associated with 94.1% protection from COVID-19 illness after 14 days following the second dose, with no cases of severe disease occurring in the vaccine arm.^37^ Similarly, 2 doses of BNT162b2 (Pfizer/BioNTech) administered 21 days apart was associated with an overall efficacy of 95% from 7 days following the second dose.^39^ Importantly, efficacy after a single dose was 52%, with protection evident as early as 12 days following the first dose. Results from additional Phase III trials are imminent.

Lower neutralizing antibody responses were induced by adenovirus-vectored ChAdOx1 nCoV-19 vaccination (AZD1222)^41^ than following the administration of mRNA vaccines, which may underlie the reduced protection, with an overall efficacy of 62.1%.^42^ Titers of neutralizing antibodies correlate strongly with protection in a number of vaccine settings.^49^, ^50^, ^51^ For SARS-CoV-2, the passive transfer of neutralizing monoclonal antibodies provided partial or complete protection of animal models in a dose-dependent manner.^10^^,^^52^^,^^53^

A key outstanding question for SARS-CoV-2 vaccine platforms is the durability of neutralizing antibody responses, for which very few data are reported so far. To date, the medium-term decay of antibody responses following vaccination has only been reported for mRNA-1273 (Moderna), in which participants were followed for 3 months after the final dose. There, group geometric mean pseudovirus neutralization ID_50_ titers declined on average –3-fold over this period, remaining between 100 and 200 (depending on the age group), just above the median of convalescent serum obtained a median of 34 days since diagnosis.^31^^,^^54^

**Figure 3:**
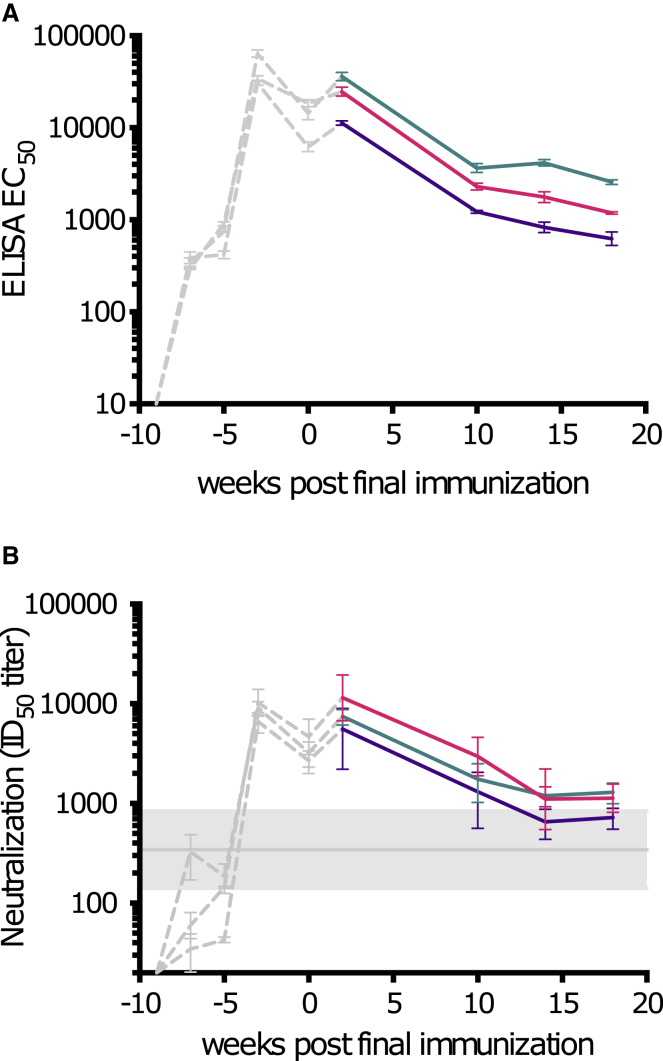
Binding and neutralizing antibody titers in rhesus macaques over 4 months following the last immunization (A) SARS-CoV-2 spike binding titers by ELISA. (B) Neutralizing antibody ID_50_ titers. Error bars depict SDs about the geometric mean for 3 replicates.

To address the question of durability, we monitored rhesus macaque binding and neutralizing antibody titers during a 4-month follow-up period after the last inoculation. Binding titers decreased on average 10-fold in the first 8 weeks following peak levels. During the subsequent 8 weeks, the decline was <2-fold (Figure 3A). This waning pattern was consistent with results observed in previous immunization studies using soluble glycoprotein immunogens, including influenza A virus hemagglutinin (HA) and HIV envelope glycoprotein (Env).^55^ Serum neutralizing potency followed a similar pattern, although the initial decline was not as steep, with just over a 4-fold decline in the first 8 weeks. Over the subsequent 8 weeks, neutralizing titers decayed <2-fold, remaining high until 18 weeks after the final immunization, suggesting a robust and long-lasting response following protein subunit immunization (Figure 3B). The geometric mean pseudovirus neutralization ID_50_ titer 4 months after the last boost was still close to 1,000 in all 3 macaques (640–1,274).

Recombinant protein subunit vaccines have been developed successfully for influenza,^56^^,^^57^ HPV, and HBV, and are also being explored for SARS-CoV-2 vaccination, including three candidate vaccines that have entered Phase II clinical trials. In a Phase I/II trial, the Novavax-developed, recombinant protein vaccine (NVX-CoV2373) adjuvanted with Matrix-M,^58^ with 2 doses administered 21 days apart, elicited extremely potent antibody responses that, at peak, exceeded the responses detected in convalescent serum, most of whom had been symptomatic, by almost 4-fold.^47^ An interim analysis from a Phase III trial suggests that this regimen afforded 95.6% protection from symptomatic infection.^44^

The efficacy of protein-based vaccines is greatly enhanced by co-administration of an adjuvant. So far, a limited number of adjuvants are approved for human studies (e.g., Alum, MF59), but several additional adjuvants are undergoing clinical evaluation, including Matrix-M, used in the present study. Consistent with results from the ongoing clinical trials with NVX-CoV2373,^47^ and with previous work,^21^^,^^59^ we found that Matrix-M adjuvanted spike protein elicited potent antigen-specific antibody responses, and that the SARS-CoV-2-spike-specific T helper cell response was Th1 biased, with the detection of interleukin-2 (IL-2) and interferon γ- (IFNγ)-producing T cells following *in vitro* recall stimulation (Figure S2).

In conclusion, here, we show in mice and non-human primates that immunization with prefusion-stabilized trimeric SARS-CoV-2 spike proteins elicits potent and long-lasting neutralizing antibody responses. Neutralizing antibody titers were exceptionally high across different immunization routes and with different adjuvants, highlighting that the SARS-CoV-2 spike protein represents a robust immunogen.

### Limitations of study

One major limitation of the present study is the lack of SARS-CoV-2 challenge. We predominantly use serum neutralizing antibody responses to assess the robustness of vaccine-induced B cell responses, as it is known that antibodies alone can protect against challenge, but the precise relationship between antibody titer and protection is not yet established. Therefore, while we show that protein immunization results in neutralizing titers that remain well above 1 in 500 for months after immunization, we cannot directly relate this to sustained protection. Furthermore, newly emerging SARS-CoV-2 variants, harboring mutations at key neutralizing antibody epitopes, have been shown to affect the neutralizing antibody potency^60^, ^61^, ^62^ and protective efficacy of some vaccines.^42^ We include neutralization assays only on the original outbreak spike protein variant and the widely circulating D614G mutant, but we have not yet assessed the neutralization potency against these new variants, which is expected to be reduced. Finally, our study was designed very early in the pandemic before anything was known about the immunogenicity of the spike protein. Recent studies^47^^,^^48^ suggest that our rhesus macaque immunization dose was probably higher than necessary to achieve robust neutralizing antibodies.

## STAR★Methods

### Key resources table

**Table:** 

REAGENT or RESOURCE	SOURCE	IDENTIFIER
**Antibodies**		

Goat anti-Mouse IgG-HRP	Southern Biotech	Cat#1013-05; RRID: AB_2794190
Goat anti-Monkey IgG-HRP	Nordic MUbio	GAMon/IgG(Fc)/PO
Goat anti-Human IgG, Fcγ	Jackson ImmunoResearch	Cat#109-005-008; RRID: AB_2337534
Goat anti-Human IgG, Fcγ-Biotin	Jackson ImmunoResearch	Cat#109-065-008; RRID: AB_2337623
Anti-CCR7 (clone G043H7) BV421	BioLegend	Cat#353207; RRID: AB_10915137
Anti-CD8a (clone RPA-T8) BV711	BioLegend	Cat#301044; RRID: AB_2562906
Anti-CD4 (clone S3.5) PE-Cy5.5	Invitrogen	Cat#MHCD0418; RRID: AB_10376013
Anti-CD45RA (clone 5H9) BV650	BD Biosciences	Cat#740608; RRID: AB_2740308
Anti-IL-13 (clone JES10-5A2) PE	BD Biosciences	Cat#562039; RRID: AB_10894004
Anti-IL-2 (clone MQ1-17H12) BV650	BD Biosciences	Cat#564166; RRID: AB_2738637
Anti-CD69 (clone TP1.55.3) ECD	Beckman Coulter	Cat#6607110; RRID: AB_1575978
Anti-CD3 (clone SP34-2) APC-Cy7	BD Biosciences	Cat#557757; RRID: AB_396863
Anti-IFNγ (clone B27) AF700	BioLegend	Cat#506515; RRID: AB_961353

**Biological samples**		

Serum from mice	This study	N/A
Plasma from NHPs	This study	N/A
PBMCs from NHPs	This study	N/A

**Chemicals, peptides, and recombinant proteins**		

Recombinant SARS-CoV-2 Spike	This study	N/A
Recombinant SARS-CoV-2 RBD	This study	N/A
SARS-CoV-2 Overlapping peptides pool	JPT peptide technologies	Cat# PM-WCPV-S
Matrix-M	Novavax AB	N/A
AddaVax	InvivoGen	Cat# vac-adx-10
Enterokinase, His, Bovine	GenScript	Cat# Z03004-500
SIGMAFAST OPD	SigmaAldrich	Cat# P9187
3,3′,5,5″-tetramethylbenzidine (ELISA TMB Stabilized Chromogen)	Invitrogen	Cat# SB02
GIBCO FreeStyle MAX Reagent	Thermo Fisher Scientific	Cat# 16447100
Lipofectamine 3000	Invitrogen	Cat# L3000075
Recombinant soluble CD40-L	PeproTech	Cat# 310-02
Recombinant human IL-21	PeproTech	Cat# 200-21
Streptavidin-alkaline phosphatase	Mabtech AB	Cat# 3310-10-1000
Staphylococcal enterotoxin B	SigmaAldrich	Cat# S4881
BCIP/NBT-plus substrate	Mabtech AB	Cat# 3650-10
Brefeldin A	Invitrogen	Cat# B7450

**Critical commercial assays**		

Cytofix/Cytoperm	BD Biosciences	Cat# 554714; RRID: AB_2869008
LIVE/DEAD Fixable blue	Invitrogen	Cat# L23105
Bright-Glo Luciferase Assay System	Promega	Cat# E2620

**Experimental models: Cell lines**		

Human: GIBCO FreeStyle 293-F cells	ThermoFisher Scientific	Cat# R79009
Human: HEK293T-ACE2	Hanke et al.^18^	N/A

**Experimental models: Organisms/strains**		

Rhesus macaque: Macaca mulatta	Astrid Fagreaus Laboratory (AFL) at Karolinska Institutet	N/A
Mouse: C57BL/6J	Jackson Laboratory	Cat# 000664; RRID: IMSR_JAX:000664

**Oligonucleotides**		

CpG B oligodeoxynucleotides	InvivoGen	Cat# trlr-2395

**Recombinant DNA**		

SARS-CoV-2 Spike plasmid	J. McLellan	Wrapp et al.^15^
Plasmid: AbVec2.0-IGHG1	Tiller et al., 2008^63^	Addgene 80795; RRID: Addgene_80795
Lentiviral backbone: pCMV delta R8.2	D. Trono	Addgene 12263; RRID: Addgene_12263
Luciferase transfer plasmid	J. Voss	Rogers et al.^10^
SARS-CoV-2 Spike plasmid with C-terminal truncation	J. Voss	Rogers et al.^10^
SARS-CoV-2 Spike D614G plasmid	J. Bloom	Crawford et al., 2020^64^

**Software and algorithms**		

FlowJo V10.7.1	Tree Star	https://www.flowjo.com/; RRID: SCR_008520
GraphPad Prism V9.0.0	GraphPad Software Inc.	https://www.graphpad.com/scientific-software/prism/; RRID: SCR_002798
Julia V1.5.3	The Julia Programming Language	https://julialang.org/

**Other**		

Strep-Tactin® XT SuperFlow® resin	IBA Lifesciences	Cat# 2-4010-010
HiLoad® 16/600 Superdex® 200 pg	Cytivia	Cat# 28-9893-35
Protein G Sepharose® 4 Fast Flow	Cytivia	Cat# 17-0618-01
His-Pur Ni-NTA resin	Thermo Fisher Scientific	Cat# 88222

### Resource availability

#### Lead contact

Further information and requests for resources and reagents should be directed to the lead contact, Ben Murrell (benjamin.murrell@ki.se).

#### Materials availability

Plasmids generated in this study will be made available on request, but we may require a completed Materials Transfer Agreement.

#### Data and code availability

The published article includes all data generated or analyzed during this study, and summarized in the accompanying tables, figures and supplemental materials.

### Experimental model and subject details

#### Ethics statement

The animal work was conducted with the approval of the regional Ethical Committee on Animal Experiments (Stockholms Norra Djurförsöksetiska Nämnd). All animal procedures were performed according to approved guidelines.

#### Mice

Twenty-four 8-and-a-half-week-old C57BL/6J mice (Jackson Laboratory - 12 males and 12 females) were used in immunization experiments. Mice were housed at the Comparative Medicine animal facility (KM) at Karolinska Institutet in individually ventilated cages. Mice had access to food and water *ad libitum* and cage enrichment included shredded cardboard and paper rolls. Cage and water changes were performed on a weekly basis and general monitoring of all mice was performed on a daily basis by technical staff. Experiments were approved by the Swedish Board of Agriculture (ethical permit number N4/16). Immunogens were diluted in sterile PBS, emulsified in AddaVax (InvivoGen) and injected subcutaneously (s.c.) in the flanks of mice at weeks 0, 4 and 7. Each arm contained 5 mice, except the low-dose RBD group which had 4. Immunization arms were balanced between males and females (with a minimum of two of each sex in each arm), and the study was not powered to detect differences in immune response due to sex. Two control mice (one male, one female) were not immunized. Tail bleeds were taken prior to each immunization and at week 10. Whole blood was allowed to clot at room temperature, and serum was separated by centrifugation, heat inactivated at 56°C for 1 hour, and stored at −20°C until use.

#### Rhesus macaques

One male and two female rhesus macaques (*Macaca mulatta*) of Chinese origin, 4-5 years old, were housed at the Astrid Fagraeus Laboratory at Karolinska Institutet. Housing and care procedures complied with the provisions and general guidelines of the Swedish Board of Agriculture. The facility has been assigned an Animal Welfare Assurance number by the Office of Laboratory Animal Welfare (OLAW) at the National Institutes of Health (NIH). The macaques were housed in groups in 14 m^3^ enriched cages. They were habituated to the housing conditions for more than six weeks before the start of the experiment and subjected to positive reinforcement training in order to reduce the stress associated with experimental procedures. The macaques were weighed at each sampling. All animals were confirmed negative for simian immunodeficiency virus (SIV), simian T cell lymphotropic virus, simian retrovirus type D and simian herpes B virus. All immunizations and blood samplings were performed under sedation with 10-15 mg/kg ketamine (Ketaminol 100 mg/ml, Intervet, Sweden) administered intramuscularly (i.m.). For macaque immunizations, stabilized spike trimer (100 μg) was mixed in 75 μg of Matrix-M (Novavax AB). Macaques were immunized intramuscularly (i.m.) with half of the dose administered in each quadricep at weeks 0, 4 and 9. Blood samples were collected pre-immunization and at weeks 2, 4, 6, 9, 11, 19, 23 and 27.

#### Cell lines

##### HEK293T cells

HEK293T (human, female) cells were used to produce lentiviral pseudotyped viruses. HEK293T cells transduced to overexpress human ACE2 (HEK293T-ACE2) were used for pseudotyped virus neutralization assays. Both HEK293T cell lines were cultured in a humidified 37°C incubator (5% CO_2_) in Dulbecco’s Modified Eagle Medium (GIBCO) supplemented with 10% Fetal Bovine Serum and 1% Penicillin/Streptomycin, and were passaged when nearing confluency using 1X Trypsin-EDTA.

##### FreeStyle 293-F cells

FreeStyle 293-F cells (Thermo Fisher Scientific), also derived from HEK293 cell line, were used for protein production, where cells were cultured in FreeStyle Expression Medium (Thermo Fisher Scientific) in a shaking incubator (135 RPM) at 37°C (8% CO_2_). All cell lines tested negative for mycoplasma by PCR.

### Method details

#### Protein production

The plasmid for expression of the SARS-CoV-2 prefusion-stabilized spike ectodomain^15^ was kindly provided by Jason McLellan. This plasmid was used to transiently transfect FreeStyle 293-F cells using the FreeStyle MAX reagent (Thermo Fisher Scientific). The spike ectodomain was purified from filtered supernatant on Strep-Tactin XT resin (IBA Lifesciences), followed by size-exclusion chromatography on a Superdex 200 in 5 mM Tris pH 8, 200 mM NaCl.

The RBD domain (RVQ-VNF) of SARS-CoV-2 was cloned upstream of an enterokinase cleavage site and a human Fc. This plasmid was used to transiently transfect FreeStyle 293-F cells using the FreeStyle MAX reagent. The RBD-Fc fusion was purified from filtered supernatant on Protein G Sepharose (GE Healthcare) and cleaved using bovine enterokinase (GenScript) leaving a FLAG-tag on the C terminus of the RBD. Enzyme and Fc-portion was removed on His-Pur Ni-NTA resin (Thermo Fisher Scientific) and Protein G Sepharose (Cytivia) respectively, and the RBD was purified by size-exclusion chromatography on a Superdex 200 (Cytivia) in 5 mM Tris pH 8, 200 mM NaCl. Proteins were re-buffered into PBS prior to immunization.

See Figure S3A–S3C for size exclusion chromatograms and SDS-PAGE analysis of purified proteins.

#### Mouse ELISAs

ELISA plates (Nunc MaxiSorp, Thermo Fisher Scientific) were coated overnight at 4°C with 100 μL of prefusion-stabilized spike protein at a concentration of 1 μg/ml in 1x PBS. After six times washing with washing buffer (0.05% Tween-20 in PBS; PBS-T), plates were blocked for 90 minutes at room temperature with 200 μL of blocking solution containing 2%(w/v) non-fat milk powder in 1X PBS and washed 6 times with PBS-T. Serum samples serially diluted in blocking solution were added and plates were incubated overnight at 4°C. Plates were washed 6 times with PBS-T, and 100 μL of a goat anti-mouse IgG horseradish peroxidase (HRP)-conjugated secondary antibody (Southern Biotech) diluted 1:5,000 in PBS-T was added to each well. Plates were washed 6 times with PBS-T, developed for 15 minutes at room temperature using 200 μL per well of peroxidase substrate (o-phenylenediamine dihydrochloride, SIGMAFAST, SigmaAldrich), and read at 450 nm in an Asys Expert 96 plate reader (Biochrom). EC_50_ titers were calculated using a Bayesian logistic curve fitting approach, allowing plate-specific minimum and maximum sigmoid parameters to account for differences between plates, and sample specific slope and offset parameters. EC_50_ titers were calculated from the posterior median value midway between the plate minimum and maximum.

#### Macaque ELISAs

ELISA plates were coated with prefusion-stabilized spike protein as described above and blocked for 1 hour at room temperature with 200 μL blocking solution containing 5%(w/v) non-fat milk powder in 1x PBS. Plasma samples serially diluted in blocking solution were added and incubated for 2 hours at room temperature. Plates were washed 6 times with PBS-T and antibody-antigen interaction was detected using 100 μL HRP-conjugated anti-monkey IgG Fc (Nordic MUbio) diluted 1:20,000 in PBS-T. Plates were washed 6 times with PBS-T, developed using 50 μL of 3,3′,5,5″-tetramethylbenzidine (TMB) substrate solution (Invitrogen) per well and stopped using 50 μL of 1M sulphuric acid per well. OD was read at 450 nm in an Asys Expert 96 plate reader (Biochrom). EC_50_ values were computed as for the mouse ELISAs.

#### Pseudotyped neutralization assays

Pseudotyped neutralization assays were adapted from protocols previously validated to characterize the neutralization of HIV^57^ but with the use of HEK293T-ACE2 cells, as previously described.^18^ Pseudotyped lentiviruses displaying the SARS-CoV-2 pandemic founder variant or D614G mutant spike protein (harboring an 18 amino acid truncation of the cytoplasmic tail) and packaging a luciferase reporter gene were generated by the co-transfection of HEK293T cells using Lipofectamine 3000 (Invitrogen) per the manufacturer’s protocols. Media was changed 12-16 hours after transfection, and pseudotyped viruses were harvested at 48- and 72-hours post transfection, filtered through a 0.45 μm filter, and stored at −80°C until use. Pseudotyped viruses sufficient to generate ∼100,000 RLUs were incubated with serial dilutions of serum for 60 min at 37°C in a 96-well plate, and then ∼15,000 HEK293T-ACE2 cells were added to each well. Plates were incubated at 37°C for 48 hours, and luminescence was then measured using Bright-Glo (Promega) per the manufacturer’s protocol, on a GM-2000 luminometer (Promega). ID_50_ titers were interpolated as the reciprocal serum dilution at which relative light units (RLUs) were reduced by 50% relative to control wells in the absence of serum using Prism 9 (GraphPad Software). Appropriate interpretation of these ID_50_ values requires linearity of the luciferase signal as the input virus decreases, which we show in Figure S3D. Statistical comparisons of pseudovirus neutralization ID_50_ titers between mouse groups were conducted with Mann-Whitney U tests, and between time points with Signed Rank tests, both implemented in the HypothesisTests.jl Julia package. Rhesus Macaque serology measurements were repeated in triplicate (with the geometric mean for the measure of central tendency, and geometric standard deviation for plotted error bars) but just once for the mouse samples, due to sample volume limitations.

#### B cell ELISpot assay

96-well multiscreen IP filter ELISpot plates (Millipore) were activated with 70% ethanol for 30 s and washed twice with 1x PBS. The plates were coated overnight at 4°C with 10 μg/ml anti-human Fcγ (Jackson ImmunoResearch). The plates were washed 3 times with 1x PBS and blocked for 2 hours with complete media. Serially diluted PBMCs were plated and incubated overnight at 37°C, 5% CO_2_. Cells were previously cultured for 72 h in complete media supplemented with 2.5 μg/ml CpG B oligodeoxynucleotides (InvivoGen), 1 μg/ml sCD40-L (PeproTech) and 50 ng/ml IL-21 (PeproTech) in 48-well plates. The plates were washed 6 times with PBS-T and incubated for 90 minutes with the following biotinylated probes: 2.5 ng/ml goat anti-human Fcγ (Jackson ImmunoResearch), 1 μg/ml prefusion-stabilized spike protein or 3 μg/ml ovalbumin to detect, respectively, total IgG and antigen-specific IgG. The plates were washed 6 times with PBS-T and incubated for 45 minutes with streptavidin-alkaline phosphatase (Mabtech AB) diluted 1:1,000. The plates were washed 6 times with PBS-T, developed with 50 μL nitro-blue tetrazolium 5-bromo-4-chloro-3′-indolyphosphate substrate (Mabtech AB) for 5 minutes in the dark and stopped by washing with sterile H_2_O. The plates were dried and spots were counted using an Immunospot analyzer (Cellular Technology Ltd.).

#### Analysis of T cell responses

Cryopreserved rhesus PBMCs were thawed and rested for 3 hours at 37°C in a 5% CO_2_ incubator. After rest, 2x10^6^ PBMCs were added per well in 96-well U-bottom plate and cultured at 37°C and 5% CO_2_ in the presence of SARS-CoV-2 S overlapping peptide pool (OLP, JPT Peptide Technologies) at 2 ug/mL or recombinant Spike trimer 57 at 10 ug/mL. For each animal, DMSO at an equal concentration to the peptide pool was used as a negative control, and Staphylococcal Enterotoxin B (Sigma-Aldrich) was used as a positive control. Ninety minutes after culture start, Brefeldin A (Sigma-Aldrich) was added to every well and the culture continued for 14-16 hours at 37°C and 5% CO_2_. The PBMCs were stained using LIVE/DEAD Fixable Blue kit (Invitrogen), followed by staining with a surface marker antibody panel (Table S1). The cells were permeabilized using Cytofix/Cytoperm solution (BD Biosciences) and stained with a panel of antibodies against intracellular proteins (Table S1). Stained cells were fixed using 1% formaldehyde and acquired with LSRFortessa flow cytometer (BD Biosciences). Data were analyzed using FlowJo software v10.7.1.

### Quantification and statistical analysis

FlowJo V10.7.1, GraphPad Prism V9.0.0 and Julia V1.5.3 were used to perform data and statistical analyses, unless otherwise stated. Statistical details of the experiments are provided in the respective figure legends or in the dedicated methods section.

## Supplemental information

Document S1. Figures S1–S3 and Table S1

Document S2. Article plus supplemental information
