# Supplementary material for: SARS-CoV-2 protein subunit vaccination of mice and rhesus macaques elicits potent and durable neutralizing antibody responses
Source: Cell Rep Med. 2021 Apr 5;2(4):100252. doi: 10.1016/j.xcrm.2021.100252 (PMC8020888; doi:10.1016/j.xcrm.2021.100252)
Supplement: Document S1. Figures S1–S3 and Table S1 [file mmc1.pdf]

Cell Reports Medicine, Volume *2*

Supplemental information

SARS-CoV-2 protein subunit vaccination of mice and rhesus macaques elicits potent and durable neutralizing antibody responses

Marco Mandolesi, Daniel J. Sheward, Leo Hanke, Junjie Ma, Pradeepa Pushparaj, Laura Perez Vidakovics, Changil Kim, Monika Àdori, Klara Lenart, Karin Loré, Xaquin Castro Dopico, Jonathan M. Coquet, Gerald M. McInerney, Gunilla B. Karlsson Hedestam, and Ben Murrell

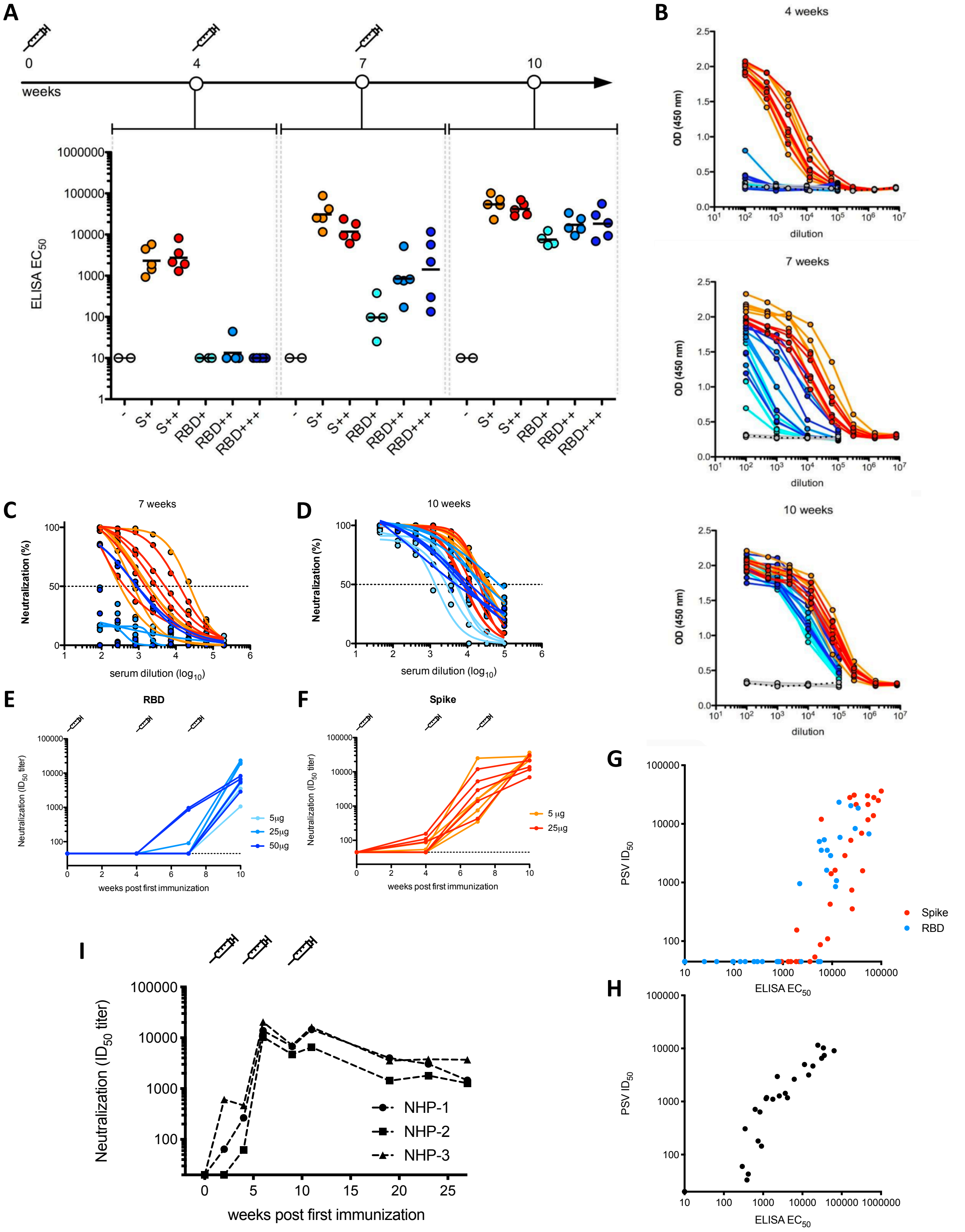

Supplemental Information Fig. 1. Serological responses to immunization of mice (A-G) and rhesus macaques (H-I). A-B. IgG antibodies detected by ELISA in immunized and control mice reveal that stabilized spike and RBD elicit antibody responses after one, two, and three doses. - (unimmunized - open circles); S+ (5 µg stabilized spike - orange); S++ (25 µg stabilized spike - red); RBD+ (5 µg RBD - cyan); RBD++ (25 µg RBD - blue); RBD+++ (50 µg RBD - navy). **C-D.** Neutralization curves for mouse sera sampled 7- and 10-weeks post-immunization. Neutralization by serum from mice immunized with RBD are displayed in shades of blue (5 µg doses, cyan; 25 µg, blue; 50 µg, navy). Neutralization by serum from

mice immunized with prefusion-conformation stabilized spike ectodomain are displayed in orange (5 µg doses) and red (25 µg). **E-F.** Longitudinal neutralizing antibody ID_{50} titers in mice immunized with RBD (**E**) or spike (**F**). Syringes depict the timing of immunizations (0-, 4- and 7-weeks). **G-H.** Correlation between spike IgG ELISA EC_{50} values and pseudovirus (PSV) neutralization ID_{50} titer in immunized mice (**G**) and macaque (**G**) sera. Neutralization ID_{50} below the limit of detection is plotted as the assay's limit of detection. **I.** Macaque pseudovirus neutralization against a SARS-CoV-2 spike with the D614G mutation showed similar neutralizing antibody sensitivity and longitudinal kinetics to that of the Wuhan 'wild-type' variant.

Extends main Figs 1 and 2.

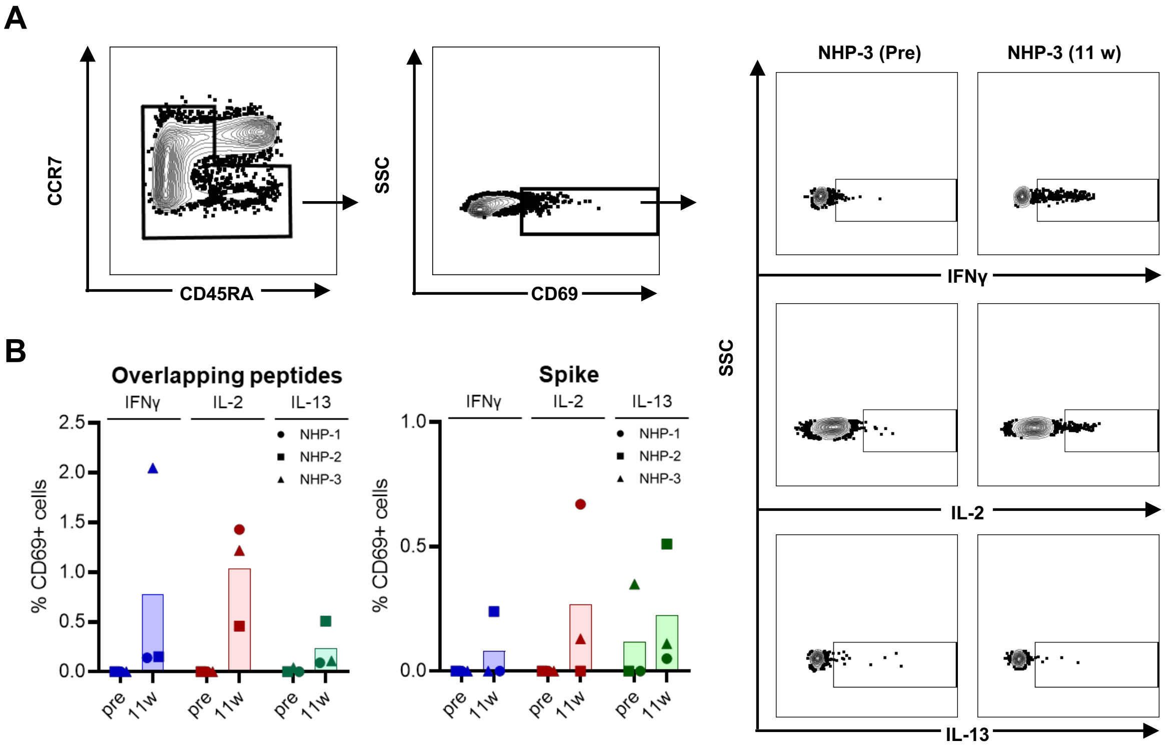

Supplemental Information Fig. 2. T helper cell responses in SARS-CoV-2 spike immunized macaques. **A.** Gating strategy displaying results from animal NHP-3 as an example. Left: PBMCs collected prior to immunization (pre) or 2 weeks after the third immunization (11w). Live, CD3+CD8- cells were gated to exclude CCR7/CD45RA double-positive naive T cells and activated CD69+ effector cells were analysed for cytokine production. Right: IFNγ, IL-2 or IL-13 cytokine staining. **B.** Summary of cytokine-producing activated effector T helper cells following stimulation with overlapping spike peptides (OLP) (left) or whole spike glycoprotein (right). Mean values for each cytokine are shown with data from individual animals superimposed.

Extends main Fig 2.

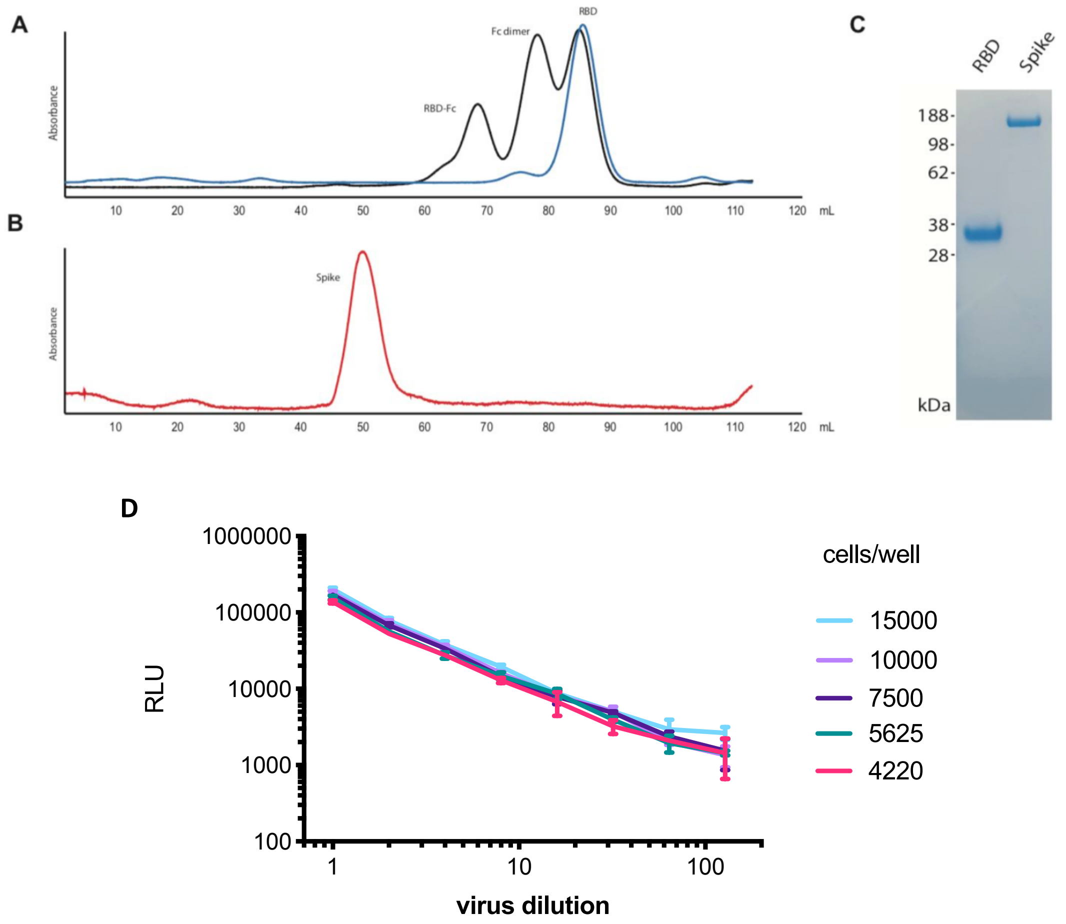

Supplemental Information Fig 3. Quality Control. A. Size-exclusion chromatograms of affinity purified RBD after enterokinase cleavage digest, before (black) and after (blue) enzyme and Fc removal. **B.** Chromatogram of affinity purified prefusion-stabilized spike ectodomain. Data from a Superdex S200 16/600 **C.** SDS-PAGE analysis of purified SARS-CoV-2 RBD and spike immunogens. **D.** Serial dilutions of input pseudovirus, and variation in target cells seeded per well, showing linearity of Luciferase signal (RLU, relative light units) in the relevant range, robust to cell count.

Linked to STAR Methods “Protein Production” and “Pseudotyped neutralization assays”.

Supplemental Information Table 1:

Marker	Fluorophore	Clone	Manufacturer
CCR7	BV421	G043H7	BioLegend
CD8a	BV711	RPA-T8	BioLegend
CD4	PE-Cy5.5	S3.5	Invitrogen
CD45RA	BV650	5H9	BD Biosciences
IL-13*	PE	JES10-5A2	BD Biosciences
IL-2*	BV605	MQ1-17H12	BD Biosciences
CD69*	ECD	TP1.55.3	Beckman Coulter
CD3*	APC-Cy7	SP34-2	BD Biosciences
IFNγ*	AF700	B27	BioLegend

*Intracellular staining

Linked to STAR Methods "Analysis of T cell responses"
